# Onsite enhancement of REEEC solar photovoltaic performance through PCM cooling technique

**DOI:** 10.1371/journal.pone.0281391

**Published:** 2023-03-10

**Authors:** Nacer Badi, Saleh A. Alghamdi, Hazem M. El-Hageen, Hani Albalawi

**Affiliations:** 1 Renewable Energy & Energy Efficiency Center, University of Tabuk, Tabuk, Kingdom of Saudi Arabia; 2 Department of Physics, Faculty of Science, University of Tabuk, Tabuk, Kingdom of Saudi Arabia; 3 Nanotechnology Research Unit, University of Tabuk, Tabuk, Kingdom of Saudi Arabia; 4 Electrical Engineering Department, Faculty of Engineering, University of Tabuk, Tabuk, Kingdom of Saudi Arabia; 5 Egyptian Atomic Energy Authority, Naser City, Cairo, Egypt; Birla Institute of Technology and Science - Hyderabad Campus, INDIA

## Abstract

The efficiency of solar panels decreases as the temperature increases and heat dissipation becomes a serious problem in hot environments such as the Arabian desert. This paper investigates the use of a phase change material (PCM-OM37P) to maintain panel temperatures close to ambient. The enhancement of the GCL–P6/60265W solar panel efficiency was demonstrated at the University of Tabuk Renewable Energy and Energy Efficiency Center (REEEC). As these solar panel arrays are remotely monitored, we were able to demonstrate the validity of our cooling solution. During peak times, a drop voltage of at least 0.6V has been realized using the PCM for cooling the PV panel. This corresponds to a cooling temperature of 5 to 6°C. This difference in operating voltages between the PCM-cooled and the reference PV panels translates into a power enhancement percentage (PEP) of about 3%. The PEP value was underestimated due to the PV string configuration where the operating electrical current is taken as the average value for both PV panels.

## 1. Introduction

Saudi Arabia has great potential for solar energy, which can be converted into electricity by using photovoltaic (PV) cells. However, due to the heat of the day, the operating temperature of the solar panel is likely to reach 60° C, resulting in a sharp decrease in solar energy performance and long-term deterioration of the solar panel. The electrical performance of solar panels is greatly affected by the operating temperature of silicon solar cells due to the properties of the crystalline silicon used in them. The efficiency decreases by 0.2–0.5% per degree Celsius, depending on the type of cells In addition to reducing the electrical performance of the PV module, overheating shortens its lifespan and harms the cells irreparably [1, 2]. In addition, the commercially offered silicon photovoltaic panels can convert only up to 20% of the incident solar energy they absorb into electrical energy, while the rest of the radiation is converted into heat [3, 4]. Heat dissipation problems are severe in hot environments such as the Arabian Desert, as the efficiency of solar panels decreases with each increase in temperature [5]. The downside of trying to cool solar panels with conventional technologies, including refrigeration or air conditioning, is that they often consume more energy than can be recovered in efficiency improvement processes. In the literature, several cooling techniques are proposed. Because of their simplicity, those based on water or air cooling are the most used [6, 7]. There are also more sophisticated mechanisms for cooling PV cells that employ phase change materials (PCM), heat pipes, impingement jets, microchannels, immersion cooling and improved heat exchangers [8, 9]. There are two types of cooling methods: passive methods that rely on natural convection or conduction and active methods that require energy to extract heat (fan, pump, etc.). In general, active cooling techniques are more efficient than passive cooling techniques. However, due to the low cost of using air or water as a cooling fluid, passive cooling may be a more appealing solution in many cases. Water is scarce in desert regions, so a dependable heat dissipation system is required to effectively cool the cells and increase their efficiency. Despite their low efficiency, air-cooling techniques based on free convection in vertical or inclined channels, such as solar chimneys, may become necessary. The latest development in photovoltaic module cooling research is hybrid cooling systems [10]. These strategies can incorporate a variety of thermal management techniques, such as passive and active cooling methods (air-water cooling, air-fin cooling, PCM-air cooling, PCM-Fin cooling, gas-liquid cooling and air-cooling with heat exchanger). Recent studies have also concentrated on coolants using two different kinds of nanofluids [11, 12] and composite phase change materials (PCM) made of two PCMs [13]. These solutions are intriguing because, thanks to the high heat storage capacity of PCMs and the high thermal conductivity of nanofluids, they result in improved heat transfer characteristics. The final objective remains the reduction of the PV module temperature and the improvement of the flexibility of the cell operating conditions.

To reduce the operating temperature, one can either use natural or induced convection to promote free cooling on the back of the panel or adjust the panel’s architecture to absorb the surplus heat. PCMs mounted on the back of solar panels are used in the latter option. PCMs are materials that, depending on their temperature, go through a reversible phase change. In the process, they absorb or reject heat. The study concept is simple: as the panels heat up, additional heat must be absorbed to completely melt the PCM.

Due to their capacity to store considerable amounts of thermal energy in comparatively tiny volumes, PCMs have become a widely used approach for latent heat storage in many applications [14]. PCM can be used in buildings to save electricity [15, 16], for storing ice [17], in hot water tanks to increase the heat capacity [18] and for thermal preservation of food (during transportation, trade, etc.), in agricultural production, dairies, greenhouses and so on [19] and finally in PV applications [20–22]. PCM was used for solar thermal applications such as thermal energy storage (TES) technology, where a photovoltaic thermal (PVT) hybrid solar collector is used to store thermal energy. Gaur et al. performed numerical studies on the thermal and electrical performance of a fully wetted absorber PVT collector with PCM as a storage medium where PCM was found to maximize the electrical and thermal performance of PVT collector [23]. A mathematical model was developed for the city of Lyon (France). During the charge phase, the heat is transferred to a storage material (PCM OM37). During the storage phase, the heat is stored in the PCM and during the discharge phase, the latent heat is transferred from the PCM to the load, a water tank household heating.

A beeswax phase change material (PCM) was used to maintain the temperature of the PV panels close to the ambient [24]. Mofijur et al. reported a review paper on PCM for solar energy usage and storage [25]. Sometimes PCM is combined with water cooling to maximize the electrical performance of such PV panels. An OM35 PCM integrated natural water circulation cooling technique was reported to allow water to flow from the bottom portion to the top and vice versa of the PCM chamber [26]. The direction of water flow from top to bottom exhibited better thermal management with a 12% reduction in the maximum top surface temperature of the PV-PCM panel.

Overall, PCM was tested for cooling purposes to face overheating in stand-alone PV panels due to excessive solar radiation and high ambient temperatures. However, there is quite a little research work made where PCM is used to cool onsite operating PV systems connected to electrical loads as in the present study. The paper is organized as follows: Section 2 presents the material and method used in the cooling process. The results and discussions are presented in Section 3. Finally, section 4 presents the conclusions of the manuscript.

## 2. Material and method

### 2.1. Solar panels

**Fig 1:**
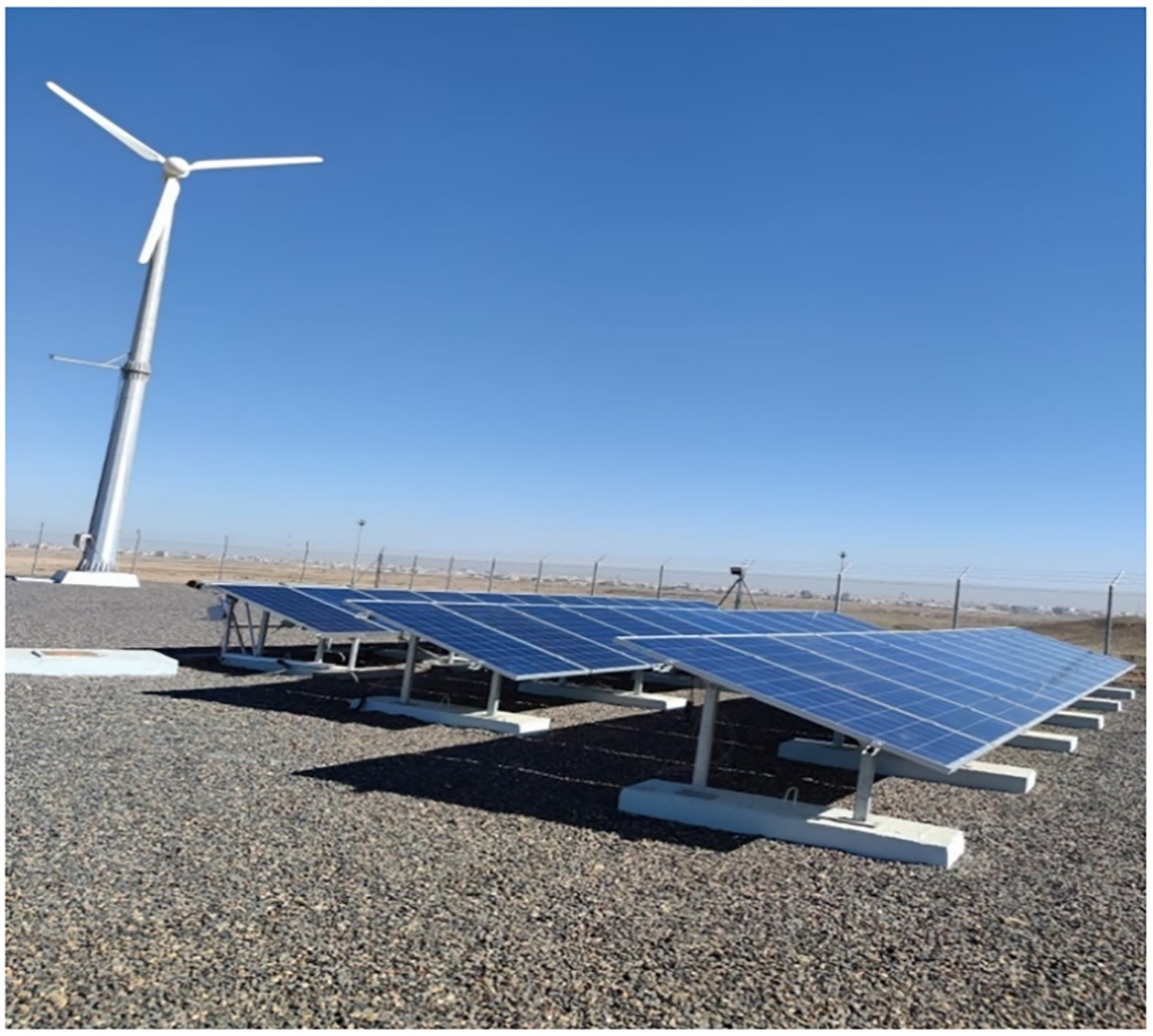
REEEC operational solar systems of the total capacity of 9Kw. **Note**: The electrical parameters are given under STC of irradiance of 1000W/m^2^, a spectrum of 1.5 air mass and a cell temperature of 25°C.

The enhancement of the PV efficiency was demonstrated at the University of Tabuk Renewable Energy and Energy Efficiency Center (REEEC) site where three identical operational solar systems with a total capacity of 9kW (i.e., 3kW each) are available (Fig 1). As these solar panel arrays are remotely monitored by REEEC, we were able to demonstrate the validity of our cooling solution. The work is conducted by using a GCL–P6/60265W solar panel, a high-efficiency polycrystalline module made by the Golden Concord Group in China. Sixty polycrystalline silicon solar cells make up the module linked in the series (6X10) with a cell dimension of 156.75×156.75mm. The electrical parameters from the GCL–P6/60265W datasheet are listed in Table 1. Our analysis data were provided by a solar monitoring station located in Tabuk and which is based on five-second measurements of electrical, physical parameters and Global Horizontal Irradiance (GHI) that hits a flat PV module. On the market, there are two main types of solar irradiance measurement. The first pyranometer type is based on the Seebeck or thermoelectric effect [27], it gives a several μV/W/m^2^ signal which is proportional to the temperature difference between a dark absorbing surface and a transparent reference surface. These pyranometers are generally much more expensive than other silicon photovoltaic pyranometers [28, 29]. But it is the most accurate and weather resistant. A thermoelectric pyranometer has been used, the device includes many characteristics suitable for the purposes, the range of measurements for our research and the geographical factors of the region. It has a range of measurement irradiance of 0-2000W/m^2^, the output signal is analog 0-5V, its sensitivity is 7–14μVW^-1^m^2^ and the operating temperature is -40℃-+80℃. A pyranometer (RK200-03) was used to measure incoming solar irradiance and was placed next to the tested solar panels. The instrument is designed to measure the solar flux density from the hemisphere above within a wavelength range of 300nm– 3200 nm. Table 2 shows the demographic position of Tabuk station, Tabuk, KSA.

**Table 1:** Electrical parameters of GCL–P6/60265W solar PV module.

Maximum power (*P*_*max*_)	265
Voltage at maximum power (*V*_*mp*_)	31
Current at maximum power (*I*_*mp*_)	8.55
Open-circuit voltage (*V*_*oc*_)	38.1
Short-circuit current (*I*_*sc*_)	9.2
Temperature coefficient of power	-0.39%/°C
Temperature coefficient of voltage	-0.30%/°C
Temperature coefficient of current	+0.05%/°C
Efficiency of the cells (%)	16.3%
Maximum operating voltage	1000VDC
Tolerance in power output	±3%

**Table 2:** The geographic position of Tabuk station, Tabuk, KSA.

Site name	Tabuk University
State	Tabuk
Latitude	28.38287
Longitude	36.48396
Elevation	781m

### 2.2. System operation

**Fig 2:**
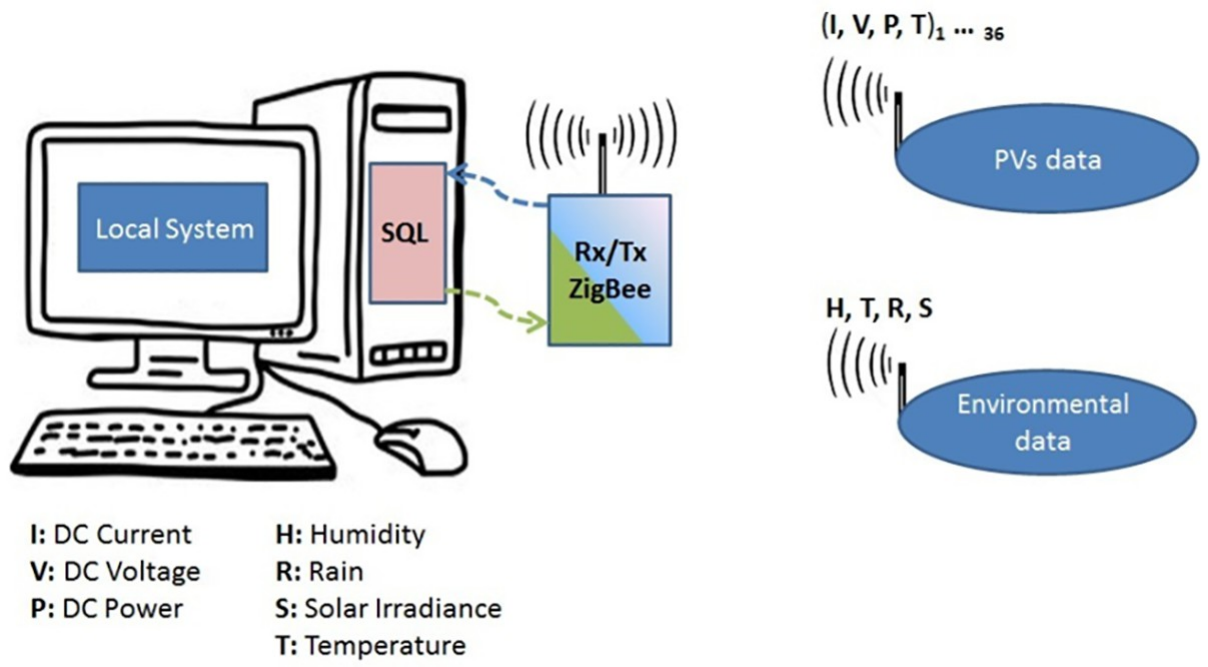
Main block diagram of the PV solar monitoring system parts.

Monitoring of photovoltaic parameters is very important for the implementation, optimization of the production process, reliability and optimal use of solar energy, as well as the lifetime and availability of all components, to reduce operation and maintenance [30–32]. Fig 2 shows the block diagram of the architecture of acquisition devices, which includes wireless sensors distributed over a solar power plant.

The wireless sensors cover various parts of the solar system such as the PV panels’ operating current and voltage, inverters, and batteries, in addition to environmental parameters such as solar irradiance, temperature, humidity, and rain [33]. In this paper, PV panel measurement units were mainly exploited. The circuit of these units used three analog sensors, they are 1) an ACS725LLCTR-10AU DC sensor with high sensitivity (264mV/A) and narrow unidirectional current sensing range (0-10A), 2) a simple voltage divider DC voltage sensor and 3) a negative temperature coefficient (NTC) sensor. Output signals of the sensors are sent to the Arduino microcontroller board (Leonardo with ZigBee Socket Module) which collects and transmits all data using the ZigBee wireless sensor network which distributes across the monitoring area of PV subsystems. Every 20 seconds, all data is automatically received by the central computer and stored in a structured query language (SQL) database.

**Fig 3:**
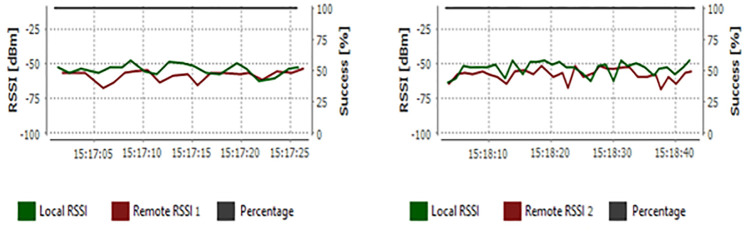
The radio range and received signal interval tests of both PV panels nodes.

The ZigBee network is based on IEEE standard 802.15.4 [34]. The 900 MHz ZigBee transceiver (TX/RX) modules with long-distance (up to 9 miles) and a 250mW power transmitter were used to install a Point to Multipoint (1 master-to-37 slave nodes) wireless sensor network WSN [33], see Fig 2. Real-time measurements of the power levels of several wireless channels are tested by the spectrum analyzer application. This analyzer has been utilized to ensure that the signal strength of the nodes used in our studies was at the required level without disruption errors, unwanted radio noise, or data loss, especially during periods of low solar panel power that supplies the electrical circuit. The Received Signal Strength Indicator (RSSI) measurements between both PV nodes and the master node are shown in Fig 3. The power values of the channels have -70 and -50 dB these levels guarantee that it does not appear as unwanted radio noise or data loss.

### 2.3. PCM cooling system

**Fig 4:**
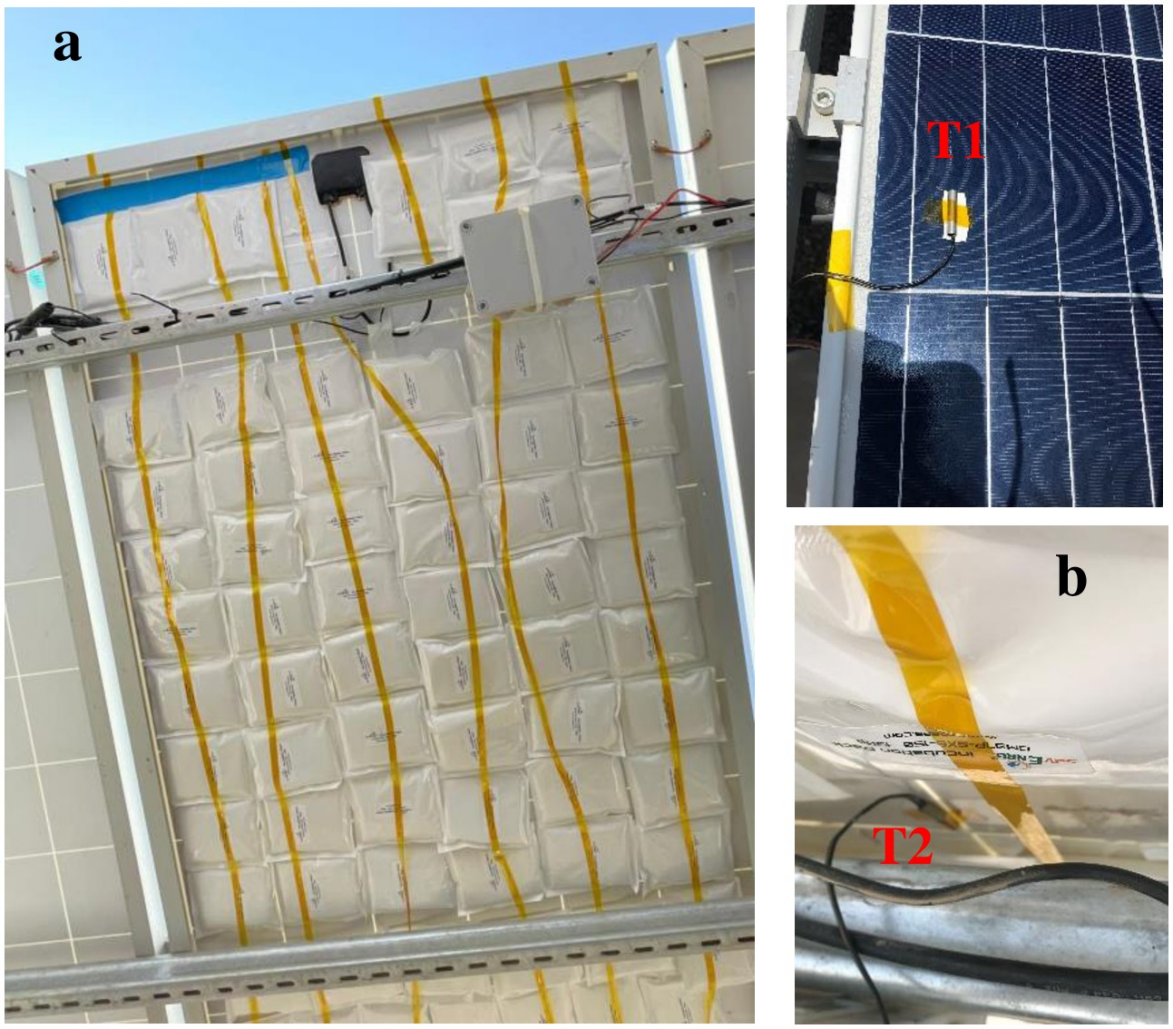
a) PCM packs loaded with PCM-OM37P material. b) Placements of the front and back thermocouples.

The tested passive cooling technique was based on a phase change material (PCM). These materials have the advantage of storing heat in the middle of the day and being able to restore it later in the afternoon or at night. These properties make it possible to better manage the recovery of the heat produced by the panels. A layer of cooling material, composed of PCM packs, was placed to fit the back of the PV panel to passively cool it and lower its operating temperature. Commercial PCM packs were purchased from RGEES–USA company under the brand name savENRG (PCM-OM37P), a mixture of organic materials which are fail-proof bio-PCM products that are designed to ensure temperature control in the range of +30 to +37 degrees Celsius. They are a unique combination of organic materials that are non-toxic, non-flammable and have a high capacity to store thermal energy in the form of latent heat. This energy is absorbed/or released at certain temperatures. PCM savENRG™ retains latent heat for thousands of cycles without any change in physical or chemical properties. PCM-OM37P consists of an ideal combination of various additives that provide a balance between solid and liquid phases at the melting point. The thermophysical properties of PCM-OM37 are shown in Table 3. The PCM packs were mounted on the back of the PV panel. The back of one GCL Solar–P6/60265W solar panel (1640mm×992mm), which is fixed on a tilted mount structure, was covered by using the BioPCM cooling packs. To reduce the thermal contact resistance in the interface area between the PV back surface area and the PCM pouch as well as maximize the heat transfer, a double-sided thermal conductive with removable adhesive on both sides was considered for the mounting of the PCM to the back of the PV panel and to increase the heat flow from the back surface of the PV panel to the PCM pouch. This special heat-resistant polyimide tape from LLP International Group is made of fiberglass with heat-conductive ceramic powder and a thermal coefficient of 1.5 W/M-K. It is highly used in the form of heat sink strips for the life support of electronic components. This way, the PV/PCM systems’ overall performance will be greatly improved, as the thermal contact resistance is expected to be reduced. In addition, the selected thermally conducive tape is highly flexible and therefore accommodates possible mechanical strain that is induced into the PV panel because of tension and compression of the PCM, without compromising efficient heat transfer. About 60 BioPCM packs (size:128 x 153mm, weight:150g, each) were held to the back of the PV panel by using a double-sided thermal conductive and strong acrylic adhesive on both sides and made of fiberglass with conductive ceramic powder, as shown in Fig 4. The Adhesive is highly strong to hold the pouches as its strength is about 360g/cm^2^. Its working temperature range is -20°C to 120°C. A second identical GCL PV panel sitting next to the first one was used as a reference for comparison purposes.

**Table 3:** Thermophysical properties of PCM-OM37.

Phase change temperature (C°)	37
Density (kg/m3)	960 (solid)
	860 (liquid)
Latent heat (kJ/kg)	218
Thermal Conductivity (W/m K)	0.16 (solid)
	0.13 (liquid)
Specific heat capacity (J/kg K)	1280

### 2.4. Operational parameters

The following performance parameters are used to evaluate the solar PV-PCM system’s performance. The electrical efficiency of a solar PV panel is given by:

$$\eta_{e}=\frac{I_{sc}V_{oc}FF}{AG}$$

where *A* is the effective surface area of the 60 cells making the PV panel and *G* is the incoming solar irradiance. The values of *I*_*sc*_ and *V*_*oc*_ are given in Table 1. The fill factor value *FF* is estimated to be 0.757. The PV panel contributes a maximum power P_max_ value of equal to *P*_*max*_ = *I*_*sc*_ · *V*_*oc*_ ·*FF*.

The improvement in PV module performance due to the addition of PCM can be evaluated. The percentage performance difference of the PV-PCM module compared to the reference PV module is defined as the power enhancement percentage (PEP) as suggested by Refs [35].

$$PEP=\frac{P_{out\;PCM}-P_{out\;ref}}{P_{out\;ref}}\times 100\%$$

where the subscripts *out ref* and *out PCM* denote the output from reference PV panel and PV-PCM panel for power efficiency, respectively.

## 3. Results and discussion

**Fig 5:**
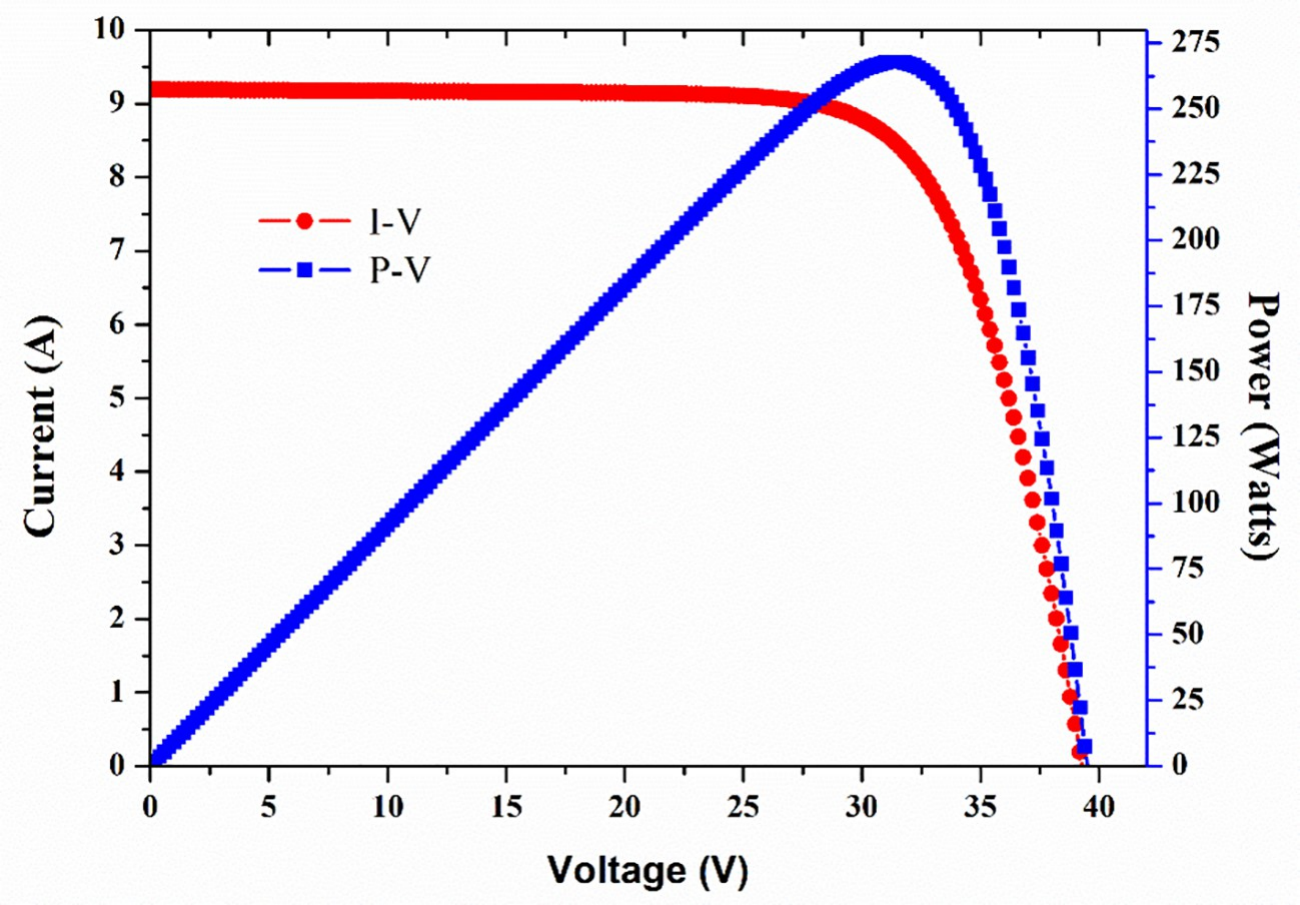
I-V and P-V characteristics of the GCL–P6/60265W PV module under STC.

First, the GCL–P6/60265W PV module’s both current and power versus voltage (I-V, P-V) characteristics are shown in Fig 5, which were obtained by using our developed unique procedure that allows accurate modeling and simulation of solar panels in the Simulink-MATLAB environment [36]. This approach is based on extracting all the necessary parameters from the data of the commercial group and evaluating the slope of the I-V characteristics under open and short conditions, as presented in Table 1.

### 3.1. Performance of PV panels in outdoor conditions

**Fig 6:**
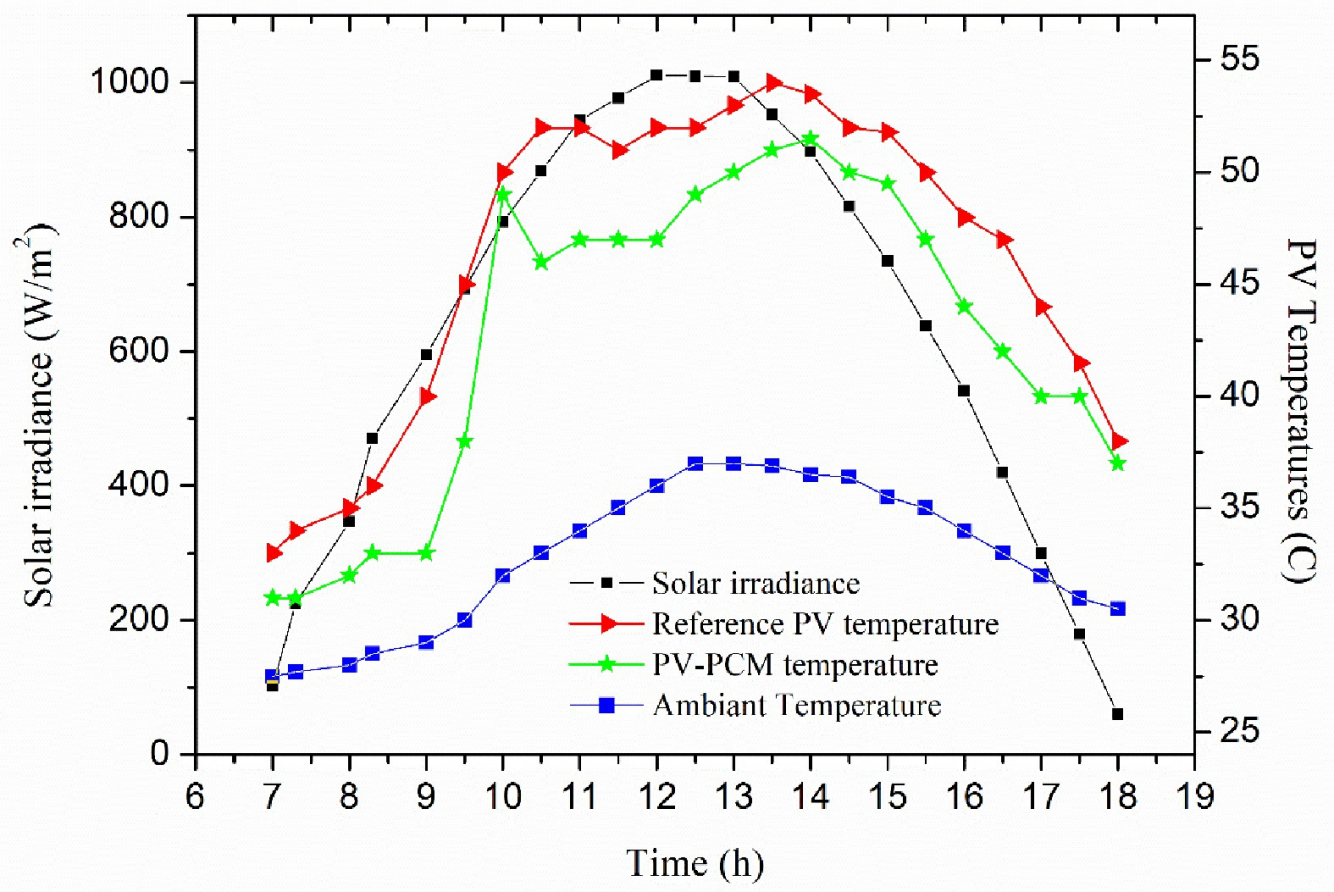
The intensity of solar radiation throughout the experimental day and variation of temperature behavior in reference and PCM-cooled PV panels.

Solar irradiance falling on a PV panel’s surface has a significant impact on its output power, its intensity during the test day is shown in Fig 6. The amount of incoming solar radiation is greatest from 11 am. until 2 p.m., which is considered the peak sun of the day. As a result, the site location can be considered to have a high potential for PV system installation [37]. The maximum amount of solar irradiance was 1010.79 W/m^2^ at 12 p.m., while the lowest amounts were 102.49 W/m^2^ and 60.31 W/m^2^ at 7 a.m. and 6 pm, which corresponds to sunrise and sunset, respectively. In a real-world scenario, the heat from the PV panel must be absorbed by PCMs throughout the day because cyclic solidification is only possible after sunlight hours. We experimentally evaluated the cooling potential of the PCM technique on the performance increase of PV panels. The effect of solar irradiance intensity on ambient and PV panel temperatures is also shown in Fig 6. High solar irradiance usually results in a high ambient temperature. As shown, the ambient temperature was 27.5°C in the early morning due to the low solar irradiance intensity of 102.49 W/m^2^. During the experimental day, the ambient temperature begins to rise as the solar irradiance rises. At maximum solar irradiance of 1010.79 W/m^2^, the maximum ambient temperature was found to be 37°C, with a daily average of 32.8°C.

**Fig 7:**
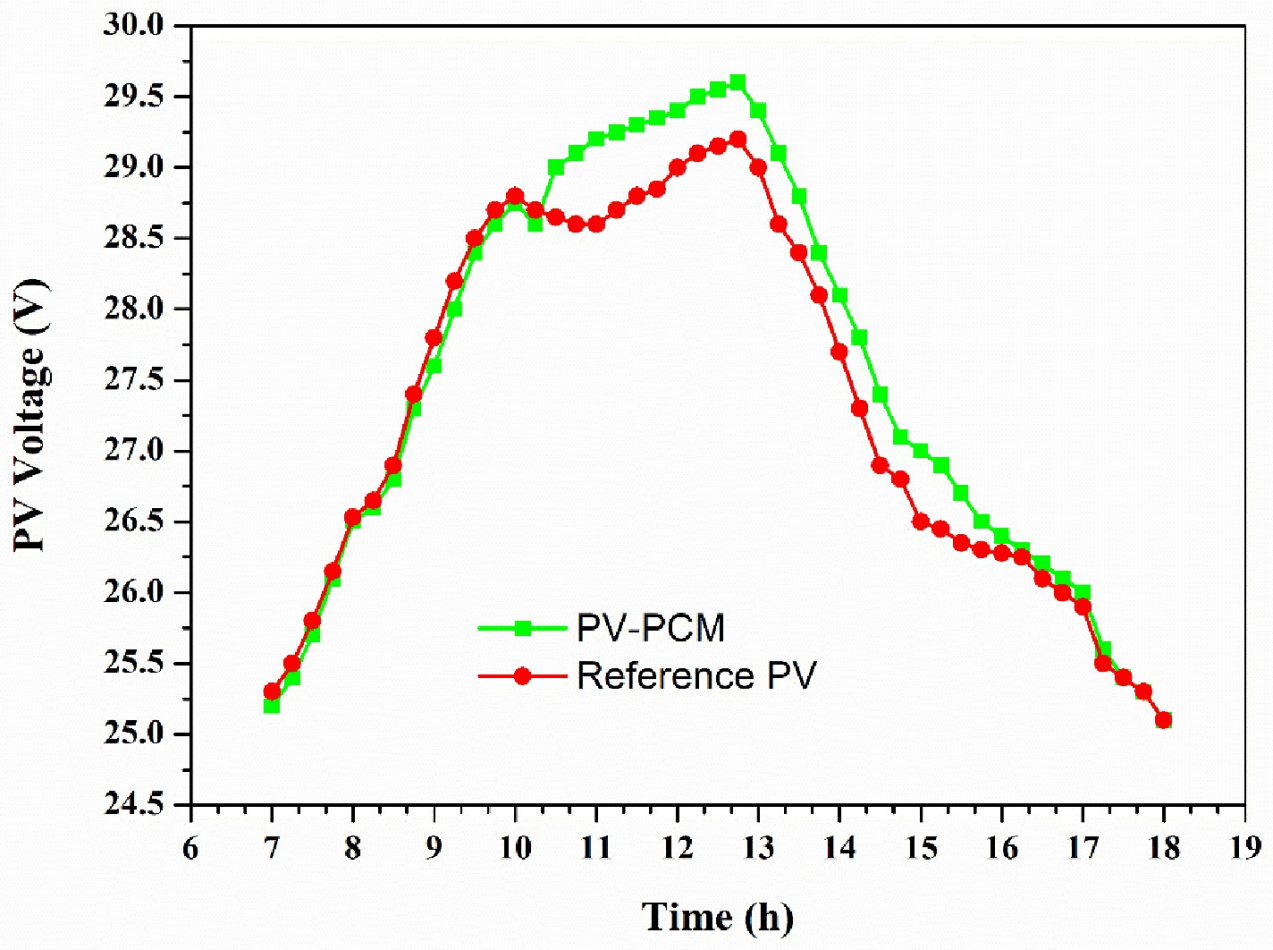
The variation of voltage behavior in reference and PCM-cooled PV panels.

During the PV operation, most of the produced solar energy happens when the PV panel temperature is higher than the ambient temperature. When the solar irradiance and ambient temperature are both increased, the temperature of the PV panel begins to rise. There is a significant difference between these two temperatures during higher incoming solar irradiance. The PV-PCM panel temperature started to increase during the phase change event, this is due to the latent heat absorbed by the PCM changing its phase. After the PCM melting process, the PCM temperature began to rise until 2:30 p.m., followed by a decrease in the PCM temperature and finally, at 6:00 p.m., the PCM temperature reached the maximum ambient temperature value of 37°C. Fig 7 shows the variation of voltage behavior in reference and PCM-cooled PV panels during the experimental day.

During peak times, a drop voltage of at least 0.6V has been realized using the PCM for cooling the PV panel. This corresponds to a cooling temperature of 5 to 6°C as determined from data in Fig 7. The operating PV panel at a lower temperature will increase its lifespan. This difference in operating voltages of the PCM-cooled and the reference PV panels also translated into a power enhancement percentage (PEP) of about 3%. This is an underestimation of PEP because in the data collection of PV string configuration where the electrical current is taken as the average value for both PV panels. The operating electrical current of the PCM-cooled PV panel is relatively higher than that of the reference PV panel. Therefore, we expect PEP to be much higher than the obtained value. Another way to enhance the overall performance of the PV panel is to combine with PCM-OM37P additional PCM materials with much higher transition temperatures nearing the highest operating temperature in Tabuk during the peak time, which is about 45°C.

## 4. Conclusion

This paper investigates the use of a phase change material (PCM-OM37P) to keep panel temperatures close to ambient. The enhancement of the GCL–P6/60265W solar panel efficiency was demonstrated at the University of Tabuk Renewable Energy and Energy Efficiency Center (REEEC). As these solar panel arrays are remotely monitored, we were able to demonstrate the validity of our cooling solution. During peak times, a drop voltage of at least 0.6V has been realized using the PCM for cooling the PV panel. This corresponds to a cooling temperature of 5 to 6°C. This difference in operating voltages between the PCM-cooled and the reference PV panels translates into a power enhancement percentage (PEP) of about 3%. The PEP value was underestimated due to the PV string configuration where the operating electrical current is taken as the average value for both PV panels. Another way to enhance the overall performance of the PV panel is to combine with PCM-OM37P additional PCM materials with much higher transition temperatures nearing the highest operating temperature in Tabuk during the peak time, which is about 45°C.

## Supporting information

S1 File (OPJ)

S2 File (OPJ)

S3 File (OPJ)
